# Revealing crosstalk of plant and fungi in the symbiotic roots of sewage-cleaning *Eichhornia crassipes* using direct *de novo* metatranscriptomic analysis

**DOI:** 10.1038/srep15407

**Published:** 2015-10-16

**Authors:** Bin Luo, Wei Gu, Jiayong Zhong, Ying Wang, Gong Zhang

**Affiliations:** 1 College of Life Science and Technology, Jinan University, Guangzhou 510632, China; 2 Key Laboratory of Functional Protein Research of Guangdong Higher Education Institutes, Institute of Life and Health Engineering, Jinan University, Guangzhou 510632, China

## Abstract

Cultivation and environmental changes can induce development of novel phenotypes in plants. For example, the root morphology of cultivated purple root *Eichhornia crassipes* differs remarkably from normal *Eichhornia crassipes* and also shows an enhanced ability to absorb heavy metal from groundwater. However, the changes in gene expression associated with these processes are unknown because of the lack of information on its large and unsequenced genome and its complex plant-rhizosphere symbiotic system. To investigate these gene expression changes, we applied a new strategy, direct *de novo* metatranscriptome analysis. Using this approach, we assembled the metatranscriptome of the entire rhizosphere and identified species-specific differentially expressed genes (DEGs) via hyper-accurate algorithms, showing a polarized plant/fungus distribution: the plant genes were responsible for morphological changes to the root system, offering a greater volume and surface area that hosts more fungi; while genes associated with heavy metal response in the fungus *Fusarium* were upregulated more than 3600-fold. These results suggested a distinct and synergistic functional response by the plant and fungal transcriptomes, indicating significant plant/fungal crosstalk during environmental changes. This study demonstrates that the metatranscriptomic approach adopted here offers a cost-efficient strategy to study symbiosis systems without the need for *a priori* genomic knowledge.

Plants coevolved with a wide range of microorganisms in their environments and have formed many different and complex patterns of symbiosis with some of these microorganisms^1^. One of the most important and widely studied of these is the rhizosphere symbiosis system, that is, the interaction of the plant root system with soil microorganisms^2^,^3^,^4^,^5^. In this symbiosis system, the plant root secretes substances that affect the behavior of microsymbionts; in turn, the microorganisms influence the physiology and metabolism of the root and can thus play an important role in the adaptation of plants to the environment^6^,^7^. The rhizosphere symbiosis system is also of considerable interest because of its potential role in remediation of environmental pollution^5^,^8^,^9^,^10^. For example, symbiotic systems can enhance the degradation of organic contamination, increase the rates of nitrogen (N) and phosphate (P) fixation, and help to sequester heavy metals in polluted water and soil^3^,^4^,^5^,^9^,^10^,^11^,^12^,^13^,^14^,^15^.

Due to the complexity of symbiotic systems and the technical difficulties in analyzing them, it has been difficult to determine the detailed mechanisms of rhizosphere interactions. The initial strategy in such studies was to investigate the plant and the microsymbiont separately: first, the ecology of the plant is characterized; subsequently, the microsymbiont is isolated and cultured under artificial conditions to investigate phylogeny, physiology, and gene functions^16^,^17^,^18^,^19^. For microorganisms that cannot be cultured, metagenomic approaches have been applied to identify the symbionts and to analyze genetic information^17^,^18^,^20^. Although both these strategies provide valuable information, they cannot be used to examine the interplay between the rhizosphere and the microorganisms. In methods where plants and microorganisms are analyzed separately, it has become clear that the gene expression profiles obtained differ from those during symbiosis^21^. Metagenomic sequencing of the entire system avoided such problems. However, due to the high complexity, data obtained from metagenomic sequencing can be difficult to analyze efficiently and to interpret, and may lead to false conclusions^17^. For example, incorrect assembly of regions from different genomes into one sequence, assembly errors due to the sequencing errors, *etc*. Another problem in analyzing plant-microorganism symbiosis is that the full genomic sequences of most plants are not available; this is a particular problem for those species that are of importance in environmental engineering. Due to the large genome size of plants, it is costly and labor-intensive to accurately sequence their genomes^22^. This creates considerable difficulties and challenges for investigations into symbiotic systems.

For non-model organisms, *de novo* transcriptome assembly can be a cost-efficient strategy; transcript fragments are produced and subsequently subjected to automated gene annotation using tools such as gene ontology and KEGG databases^23^,^24^,^25^. Short sequencing reads are then mapped to the assembled fragments to determine transcript quantities^26^. From such information, it is possible to infer connections between gene expression profiles and phenotype^27^,^28^. However, close symbiotic relationships can make it difficult to separate plant RNA from microsymbiont RNA. Given the complex nature of symbiosis systems and the errors in the sequencing datasets, transcript assemblies may contain flaws. In such cases, the standard algorithms are not effective and large amounts of mappable reads can be lost; such problems can bias the quantification and create many false positives and false negatives in gene identification^29^,^30^.

In this study, we adopted a strategy we term “direct *de novo* metatranscriptomics” to investigate the rhizosphere symbiosis system of the water hyacinth *Eichhornia crassipes*. Water hyacinth is highly infected by vesicular-arbuscular mycorrhizas^31^. The symbiotic microorganisms may play a considerable role in cleaning chemical pollutants, *e.g*. ethion^32^. Among the symbiotic microorganisms, fungi population is one order of magnitude higher than bacteria, indicating that the fungi might be a primary functional player in the symbiont^33^. In cultivation, the plant can be induced to produce a purple root (PR) phenotype in which the roots swell significantly and darken in color^34^,^35^,^36^. This phenotype is of considerable interest as many studies have reported it possesses an enhanced ability to remove organic and heavy metal contaminants from water, compared to normal *E. crassipes* (NR)^33^,^37^,^38^. Unfortunately, the genomic sequence of *E. crassipes* (and of other members of this family) has not yet been determined; consequently, standard approaches to analyze changes in symbiotic interactions after the induction of the PR phenotype cannot be applied. In our strategy of direct metatranscriptomics, we treat the symbiotic system as a single entity and directly assemble the transcripts from both plant and microsymbionts using next-generation sequencing reads. Assignment of the transcripts to species is then performed after assembly and functional annotation, and is based on sequence alignment with the aid of rDNA ITS-DGGE (denaturing gradient gel electrophoresis for the internal transcribed spacer) and ITS metagenome sequencing. We demonstrate here that this strategy (outlined in Fig. 1) provides a direct and effective way to investigate functional gene expression profiles in complex symbiotic systems under different environmental conditions and without any *a priori* knowledge of genomic sequences.

## Results

### Overview of the strategy

**Figure 1:**
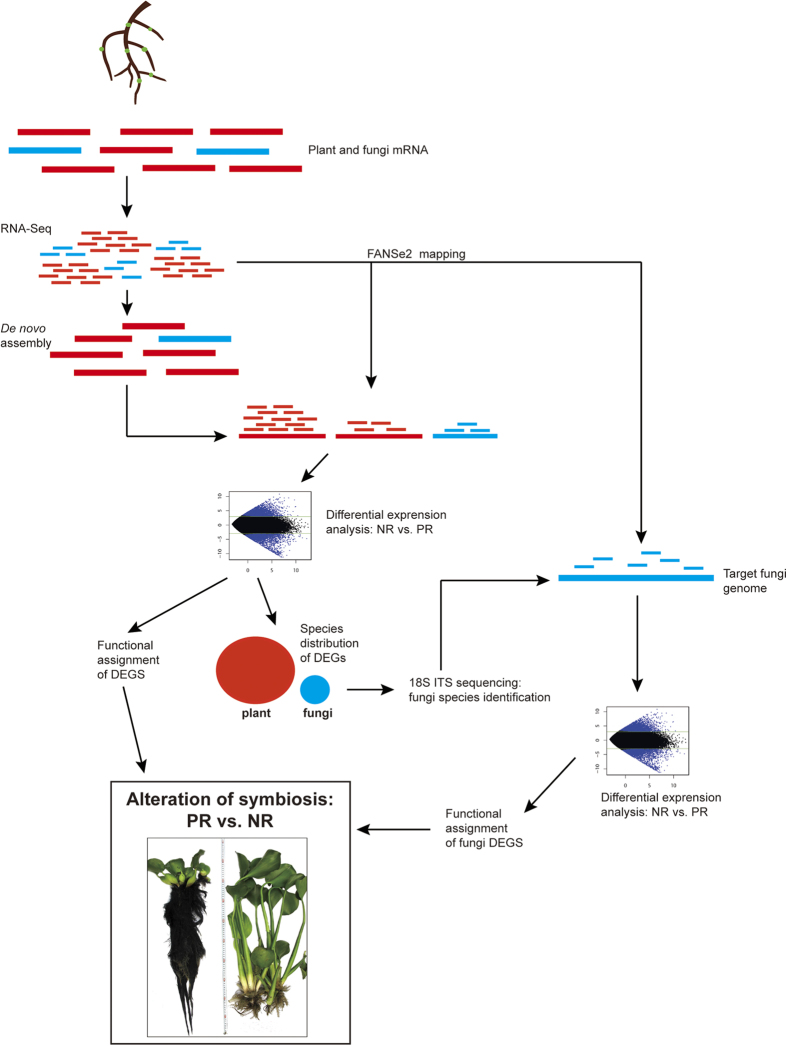
An outline of the strategy for direct *de novo* metatranscriptome analysis of the changes to the rhizosphere of two types of *Eichhornia crassipes*: normal plants (NR) and purple root plants (PR). In the illustration, red items represent sequences from the plant, and cyan items represent sequences from fungi.

An overview of the protocol used here is provided in Fig. 1. We considered the whole rhizosphere as a single system. Total mRNA was collected from the rhizosphere, fragmented, and sequenced in an Illumina HiSeq-2500 sequencer to obtain millions of short reads. The reads were assembled into contigs representing mRNA species, including plant mRNAs and fungal mRNAs. Then, the sequenced reads were mapped onto the assembled contigs to determine the quantity of each mRNA. Differentially expressed genes (DEGs) from NR and PR plants were aligned to known gene sequences of all species to identify their origin (plant or fungal) and their potential functions.

To investigate fungal transcripts more thoroughly, internal transcribed spacer (ITS) sequences from rDNA were amplified from rhizosphere genomic DNA and sequenced to confirm the identities of the fungal species that showed significant changes in PR. The mRNA-seq reads were again mapped to the genomic sequences of these fungal species to more accurately quantify the DEGs. Their functional annotations were then used to assess their contribution to the entire system.

Inevitably, the assembled contigs contain flaws^39^; therefore, the mapping algorithm needs to be accurate and error-tolerant to minimize bias in quantification. Additionally, the mapping algorithm should also be very sensitive in order to distinguish mRNA reads originating from each species in a complex metatranscriptomic mixture. Hence, we chose to use the FANSe2 mapping algorithm here^30^; this algorithm has been shown experimentally to have the minimal false positive and false negative identifications and the minimal quantification bias^30^,^40^.

### PR and NR differ in phenotype and function

**Figure 2:**
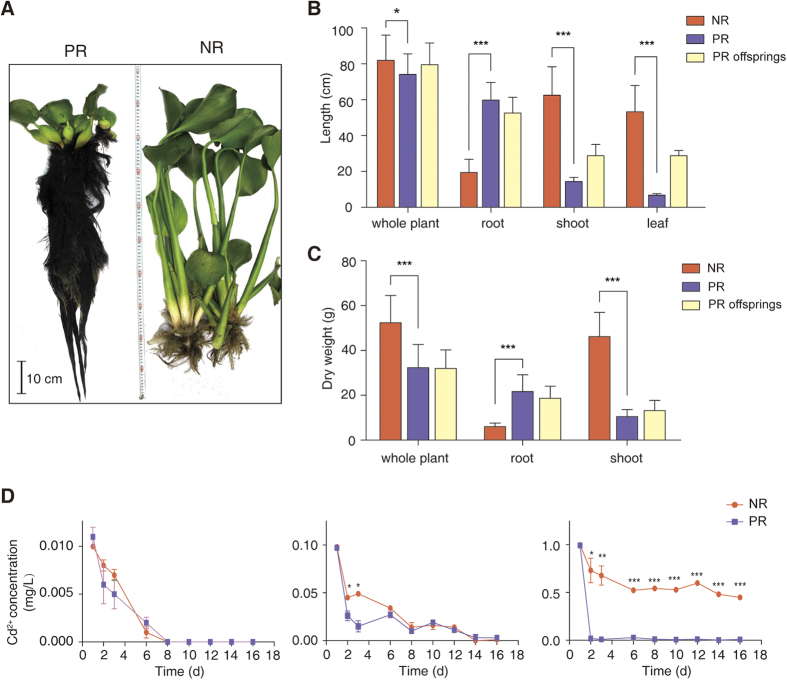
Phenotypic and functional differences of PR and NR. (**A**) Typical NR (right) and PR (left). The plants were photographed simultaneously with a scale in the middle. (**B**,**C**) Comparison of morphological traits of NR, PR and PR offspring grown in clear water: lengths of different tissues (**B**); and dry weights (**C**). The data are reported as mean ± standard deviation. (**D**) Rates of absorbance of Cd^2+^ ions by NR and PR plants cultured in water with 0.01, 0.1 or 1 mg/L Cd^2+^ at day 1 (left, middle and right panels, respectively). Cd^2+^ concentrations were monitored for 16 days. One, two and three stars represent *P* < 0.05, *P* < 0.01 and *P* < 0.001 (two-tailed *t*-test), respectively.

The morphology of the roots of NR and PR differ dramatically (Fig. 2A). The roots darkened and significantly expanded by over 3-fold in length and 3.5-fold in dry weight (Fig. 2B,C). In contrast, the above-water tissues shrank significantly by about 4.6-fold on average (Fig. 2B,C).

We tested the relative abilities of the NR and PR to remove pollutants from water. Both groups of plants reduced Cd^2+^ concentrations (Fig. 2D). At a Cd^2+^ concentration of 0.01 mg/L, the rate of absorption in both plant groups was similar. At a higher Cd^2+^ concentration (0.1 mg/L), the PR showed a higher rate of absorption than NR. At the highest tested concentration of Cd^2+^ (1 mg/L), the PR had removed 99% of the heavy metal by the second day, while the NR had only absorbed half of the pollutant after 16 days. Overall, the PR showed a higher rate of absorption of Cd^2+^, particularly at higher concentrations of the metal.

Interestingly, these prominent ecological phenotypes were not efficiently inherited by the offspring of plants with induced PR, which showed partial reversion to the wild-type morphology (Fig. 2B,C). As water hyacinth mostly conducts vegetative propagules and prolific clonal reproduction, ~80% of introduced populations were composed of a single clone^41^. Therefore, we expect no genome difference between PR and NR. Indeed, we found no sequence variations in the intergenic regions of the chloroplast *psbA-trnH*, *trnL-trnF* or the rDNA ITS sequences in NR and PR plants, or in the offspring of PR plants. These results indicate that altered gene expression profiles and not genetic mutations in the plant and/or rhizosphere microbiome dictated the phenotypical and functional changes. To further investigate the changes in gene expression, we next performed metatranscriptome sequencing.

### Sequencing and metatranscriptome *de novo* assembly

We extracted the total RNA from the entire root and isolated the polyA+ mRNA to next-generation sequencing. This would simultaneously provide transcriptome information for both plant and fungi. We obtained 76.7 million 100 nt pair-end clean reads from the mRNA sequencing of NR and PR (the statistics of the datasets are listed in Supplementary Table S1). We applied two widely used assembly tools, namely Trinity and Velvet, to perform metatranscriptome *de novo* assembly. The optimal results of the two algorithms for the datasets are shown in Table 1. Trinity yielded 3 ~ 4-fold longer N50 and a longer maximum contig length than Velvet (Table 1 and Supplementary Figure S1). Comparison of the contig length distributions also indicated that Trinity was better in assembling short reads into longer contigs than Velvet (Supplementary Figure S1). Longer contigs represent more continuous mRNA information and facilitate functional annotation by homology. Therefore, Trinity performed better in this metatranscriptome assembly, and we used its results in all further analyses.

A potential risk of such a powerful *de novo* assembly tool is that it might erroneously assemble two fragments that actually originated from two different mRNA species (false fusion)^17^. This kind of mistake is more likely in cases with low sequencing quality (which is not the case here, Supplementary Table S1) or in cases with high complexity in the system. To examine this risk, we aligned contigs longer than 300 nt to the NCBI non-redundant database using the Blastx tool. We found that 76% of the transcripts from NR and 70% from PR were homologous to known transcripts in the database (Table 1). By comparison, previous *de novo* transcriptome assemblies in the Chinese fir^42^ and in *Nomuraea rileyi*^43^ yielded 58.3% and 65.3% of contigs with homology to the database, respectively. Therefore, our results here showed better homology and potentially had a lower risk of false fusion. Using the more stringent criteria of >50% identity and >50% coverage, we still found that 50.4% and 41.7% of our contigs were homologous to the NCBI non-redundant gene database for the two groups, respectively. This analysis provides a solid base for the downstream functional annotation.

To confirm that most of the mRNA-seq reads were utilized in the assembly, we mapped the short reads onto the assembled contigs using the FANSe2 algorithm, allowing 2 mismatches. This is a very stringent criterion for 100 nt reads, since the base-calling step itself can cause >2% error^44^. We found that 86.50% of the reads from the mRNA-seq dataset of NR and 86.68% from PR mapped to the contigs, showing that the assembly utilized most of the reads.

### Differential gene expression analysis

**Figure 3:**
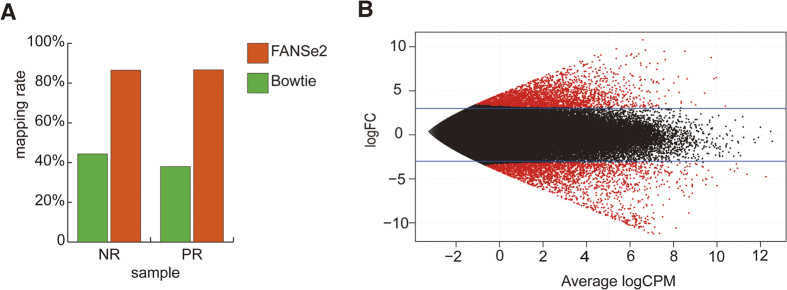
Mapping rate and Volcano plot of gene expression differences. (**A**) Mapping rates produced by FANSe2 and Bowtie. (**B**) Differential gene expression between NR and PR.

To identify the DEG profiles caused by the PR cultivation, we mapped the reads to the assembled contigs. The read counts of each contig are listed in Supplementary Table S2. In addition to the FANSe2 algorithm mentioned above, we also tested the widely used Bowtie algorithm. Bowtie mapped less than half of the reads compared to FANSe2 at the same error allowance (Fig. 3A) showing that FANSe2 is more robust for mapping reads in such a complex metatranscriptomic system.

On the basis of the dramatic phenotypic differences of NR and PR, we anticipated large-scale changes in gene expression. We applied the edgeR package to evaluate the DEGs. Under the criteria of |log_2_fold-change (log_2_FC)|≥3 and FDR < 0.01, we identified 2838 upregulated contigs and 2394 downregulated contigs (Fig. 3B).

### The functional and phylogenic analyses of DEGs

**Figure 4:**
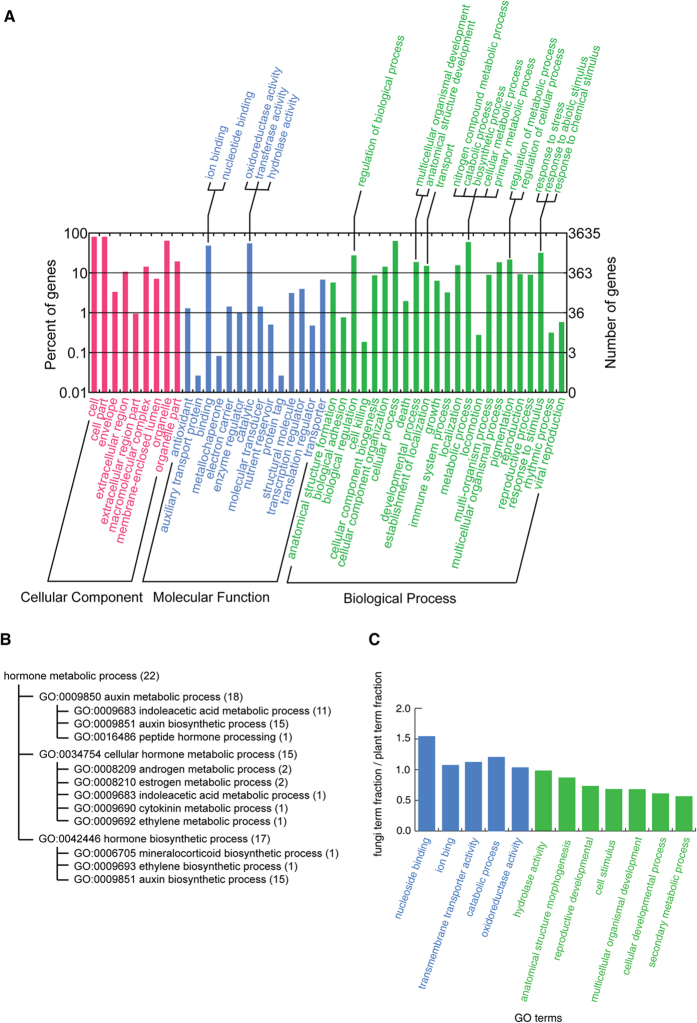
Gene ontology analyses. (**A**) Gene ontology classification of assembled transcripts showing the top three levels of terms from PR. Second levels terms are listed below, and selected third-level terms are listed above the diagram. (**B**) GO assignments under the GO term “hormone metabolic process”. Numbers in brackets indicate the number of GO assignments of each GO term. (**C**) The ratio between fungal GO term fractions and plant GO term fractions of the annotated DEGs. For details please see the main text and Supplementary Table S3. Ratios larger than 1 indicate functions that are carried out by fungal genes, and *vice versa*.

All DEGs were subjected to BLAST2GO analysis. In total, 3606 differentially expressed contigs were assigned with gene ontology (GO) annotations (Fig. 4A). In the GO category Molecular Function, the DEGs clustered in the terms “binding” and “catalytic”. More specifically, most DEGs concentrated in “binding” functions, including nucleotide binding, ion binding, and metal cluster binding; these terms correspond to global transcriptional regulation and metal-ion binding and metabolism. In “catalytic” functions, the largest numbers of DEGs were involved in oxidoreductase activity, transferase activity, and hydrolase activity, corresponding to stress responses against the potential oxidative stress caused by a heavy metal^45^, suggesting PR’s enhanced potential to counteract possible heavy metal in its environment. In the GO category Biological Process, the DEGs were mostly clustered in the terms “cellular process” and “metabolic process”. These terms were particularly prominent in DEGs from PR and may be associated with the higher metal uptake potential in these tissues. Under “cellular process”, the DEGs were mostly enriched in the term “cell cycle”. More specifically, the GO terms “M phase of mitotic cell cycle”, “regulation of mitotic cell cycle”, “reciprocal meiotic recombination”, “cell cycle phase” and “cytokinesis during cell cycle” were enriched (Supplementary Figure S2). These results indicated that the cell cycle is accelerated, which might explain the expansion of the root.

We next subjected the annotated DEGs to KEGG pathway analysis and found that 2571 DEGs were present in KEGG pathways. The pathways with the greatest up- or down-regulation of DEGs are listed in Table 2. The plant hormone signal transduction pathway was the most upregulated pathway and was not included in the downregulated list, indicating that this pathway is highly activated in PR. In addition, the GO term “hormone metabolic process”, which consisted of 22 up-regulated DEGs, showed concentrated DEGs on auxin (indolacetic acid) biosynthetic process (Fig. 4B). As auxin (indolacetic acid) is known to accelerate root growth in a certain range of concentration, this outcome was consistent with the drastic morphological changes in the whole plant in PR, and also in concordance with the gene ontology analyses. Plant-pathogen interaction pathways were both up- and downregulated, indicating that the interplay between the plant and symbionts shifted following the PR cultivation. The cell cycle pathway contained 43 upregulated DEGs, indicating that the cell cycle was accelerated. Possibly, this accounts in part for the dramatic morphological expansion of the roots. The metabolic pathways for sugars and their derivatives were upregulated; this was consistent with the requirement for biological functions that provided more biomaterials and energy for root expansion. Ribosome-associated genes were downregulated, indicating that translation might have been suppressed. In many organisms, such as yeast, humans, and plants, inhibition of the translation machinery has been shown to be an effective mechanism for adaptation to heavy metal induced stress^46^,^47^,^48^,^49^. In sum, the results of the KEGG analyses were consistent with the phenotypic changes, indicating the effectiveness of our metatranscriptome sequencing and annotation.

To separate pathway alterations in plants from those in the symbiotic fungi, we estimated the relative likelihood of the GO terms to be carried by plant-like genes or fungi-like genes. To minimize bias, only GO terms with more than 100 annotated DEGs were selected. We then calculated the fractions of GO terms for plant-like genes (transcripts aligned better to plant genes) and fungi-like genes (transcripts aligned better to fungal genes). The ratios of the two fractions for each GO term were calculated (Fig. 4C and Supplementary Table S3): a ratio >1 indicated that this function was more likely carried out by fungi, and *vice versa*. Based on this criterion, we found that nucleoside binding, catabolic process, transmembrane transporter activity, ion binding, and oxidoreductase activity were more likely to be carried out by the fungi. These are the functions that are crucial to heavy-metal binding and processing pathways. The remaining GO terms, including structure morphogenesis, developmental processes, and secondary metabolic processes, were more likely to be carried out by plant genes. This indicates that PR expanded its root system adjusted its metabolism in a way that offered a better hosting environment for fungi. The cultivation of PR caused a large expansion in the fungal population associated with the plant and in the metabolic processes in these symbionts as they expanded and changed to provide a new ecological function.

### Analysis of changes in symbiont fungal species between PR and NR

To examine the change in the symbiotic fungal species between PR and NR and to identify the species capable of processing a heavy metal, we amplified the rDNA ITS sequences of all eukaryotes using specific primers from equal biomass of roots, and then analyzed the products using PCR-DGGE and next-generation sequencing methods.

**Figure 5:**
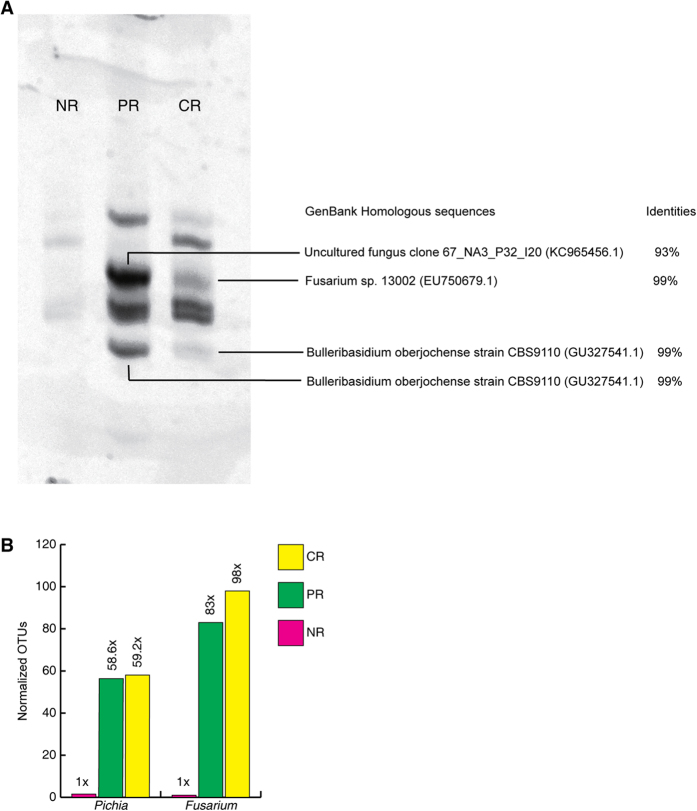
Identification and quantitation of the rhizosphere fungal species. (**A**) PCR-DGGE of rDNA ITS sequences. The marked bands were excised from the gel and sequenced by capillary sequencing. The sequences were aligned with the NCBI GenBank database to determine the species. (**B**) Normalized OTUs of the fungal species *Pichia* and *Fusarium* in NR, PR and PR grown in contaminated water (CR).

PCR-DGGE yielded considerably more intense bands in samples from PR than NR. The amplification products were derived from genomic DNAs obtained from identical biomasses of NR and PR root tissues. The more intense bands from PR suggested they hosted much larger amounts of symbiont microorganisms in the same root biomass. Considering much higher root biomass of PR than NR, a single individual of PR hosts remarkably more fungi. The PCR-DGGE analysis of PR incubated in water containing 1 mg/L cadmium (CR) also yielded more intensely stained bands than seen in NR; i.e. growth in water containing cadmium appeared to alter the populations of symbiotic fungi. We excised two significant bands that appeared in the CR group but not in NR and sequenced these using capillary sequencing technology (Fig. 5A). After alignment with the NCBI database (fungal ITS sequences), these two bands were identified as being derived from the fungal genera *Fusarium* and *Bulleribasidium*. Bands at the corresponding positions from PR grown under normal conditions were also excised and sequenced; they were identified as an uncultured fungal species and *Bulleribasidium* (Fig. 5A). *Bulleribasidium* decreased considerably in water containing cadmium; therefore, it is unlikely to be an important species with respect to environmental remediation. Due to the limited resolution of DGGE, only *Fusarium* was identified as potentially useful for remediation purposes.

To enhance the resolution of species identification and improve quantification, we carried out next-generation sequencing on the rDNA ITS sequences using an Illumina MiSeq sequencer running at 2 × 250 nt mode. We obtained 0.12 M ~ 0.26 M reads from each sample (Supplementary Table S4) and identified 118 ~ 152 genera from each sample. The operational taxonomic unit (OTU) count was used to quantify each genus, i.e., to estimate their proportional representation (Supplementary Figure S3). To obtain absolute numbers for each species in the samples, we normalized the OTUs using the qPCR quantification of *Fusarium* (Supplementary Table S5). We anticipated that a pollution remediating species would increase in PR compared to NR, and increase further in the presence of contaminated water. Therefore, we set the criterion that the normalized OTU in PR was at least 50-fold elevated compared to NR and that it was further increased in plants in contaminated water. Only two genera, *Pichia* and *Fusarium*, fulfilled this criterion; *Fusarium* showed the greatest level of increase (Fig. 5B). Notably, *Fusarium* was the only genus that were spotted by both methods, indicating that *Fusarium* is most likely responsible for the heavy metal absorbance. In support of this indication, literature showed that Fusarium exhibits strong ability to absorb heavy metals, e.g. uranium, copper, thorium, etc^50^,^51^,^52^.

### Differential gene expression in Fusarium and the relevance to the heavy metal absorption

The major ways of fungi heavy metal absorption in rhizosphere are enhanced transporter activity and/or enhanced metal binding/chelating (reviewed in^53^). We next addressed the genes that might be responsible for these functions. We used the genomic sequence of *Fusarium verticillioides* (GenBank Assembly ID: GCA_000149555.1) as the reference genome and mapped the mRNA-seq reads of the NR and PR to this reference genome. Seven upregulated DEGs were identified using the edgeR package, and 4 genes were annotated by BLAST2GO (Table 3). Contig62 was annotated as a protein that chelates multiple metal ions. The edgeR package showed that this gene was upregulated 10.6-fold in PR in the same number of Fusarium cells. Considering the 98-fold increase of Fusarium population and 3.5-fold increase in root biomass in PR, the metal ion binding gene contig62 was up-regulated for 3600-fold in PR, which might explain the heavy-metal absorption capability of the roots of PR, even before the application of heavy metal. Meanwhile, the stress response gene contig91 was upregulated 3-fold, implicating that this fungi is ready to counteract the stress created by the heavy metal.

## Discussion

### Direct metatranscriptomic analysis: the algorithm makes a difference

Metatranscriptomic *de novo* assembly of poly(A)+ mRNAs has been used previously to study fungal communities, particularly in soil. A small number of studies have also exploited this approach to investigate rhizosphere symbiosis systems with available plant genomes^54^. However, as the genomes of most plants are unsequenced, the complexity of the plant-fungi metatranscriptome poses considerable challenges for analysis. Evolutionarily diverse eukaryotic species, such as plants and fungi, share some degree of homology in their mRNAs. This can interfere with efficient assembly, especially from the short reads generated by Illumina sequencers, and can result in production of relatively short contigs and of single-nucleotide errors in these contigs. Therefore, it is essential to employ *de novo* transcriptome assembly algorithms such as Trinity, and mapping algorithms with high error-tolerance, accuracy, and robustness such as FANSe2 to efficiently map the reads to the assembled contigs and to identify DEGs, as shown in this study. Less robust and less accurate algorithms, such as Bowtie, fail to map most of the reads to the assembly and are thus less suitable for DEG identification. This factor may have hindered the success of previous studies.

Although the *de novo* assembly in this study was limited by the short reads, the contig lengths may be largely improved by third-generation sequencing, such as PacBio SMRT sequencing that can yield 3 ~ 30 kb reads, when the throughput catches up with the current second-generation sequencers in the future. However, the 12 ~ 17% error rate of SMRT sequencing again necessitates highly accurate and error-tolerant algorithms to map the short reads onto the contigs for gene expression quantification or correction of the assembly. We have shown that highly accurate FANSe series algorithms tolerate ~20% deviation in the reference sequence, while the previous algorithm gives numerous false positives and false negatives^40^. Therefore, our strategy may be directly employed in the era of third-generation sequencing.

In summary, the protocol adopted for this study shed lights on the selection of the appropriate algorithm for *de novo* metatranscriptomic studies.

### Polarized but interconnected functions of plant/fungal DEGs

The identified DEGs were classified as plant or fungal genes according to sequence homologies. These genes showed polarized function assignments: the plant genes were responsible for creating the increased volume and surface to accommodate symbionts; the fungal genes regulated metabolic functions related to the environment. Our DGGE and rDNA ITS sequencing analyses also showed a clear and dramatic taxonomic shift in the fungal species in PR and growth in water contaminated with a heavy metal. These results demonstrated that environmental changes effectively favored particular species of symbiotic fungi. Most current studies focus on the functions of bacteria in the rhizosphere microbiome^55^,^56^; our results here indicate the necessity of including fungal species when studying the environmental and ecological functions of plants, especially for aquatic plants.

Notably, plant hormone signal transduction and plant-pathogen interaction were the most significant functions carried out by DEGs, indicating a profound and complex crosstalk between *E. crassipes* and its symbiotic fungi. This crosstalk may function in the taxonomic selection of symbiotic fungi flora, and the detailed mechanism warrants further investigation. The dominance of plant-pathogen interaction functions in DEGs implies that the taxonomic shift is unlikely to be the consequence of succession with respect to competition, at least in this instance. This scenario is different to the succession of rhizosphere bacteria in soil, suggesting a distinct difference in the behavior of rhizosphere fungal species and indicating a multifaceted rhizosphere system with a complex nature and interactions among plant, fungi, and bacteria.

## Conclusions

We demonstrated for the first time the feasibility of using direct *de novo* analysis of the metatranscriptome to investigate the *E. crassipes* rhizosphere without any *a priori* knowledge of the plant genome. In addition to *de novo* metatranscriptome assembly, we showed that use of a robust, error-tolerant and accurate mapping algorithm overcame flaws in the assembled contigs and enabled the identification of DEGs. The plant and fungal DEGs exhibited distinct and synergistic functional patterns. During the cultivation or exposure to environmental change, significant crosstalk was observed between plant and fungal genetic pathways, which may be the causal factor for the changes in the symbiotic fungi and the development of metabolic pathways capable of dealing with an environmental pollutant. This strategy can be also expanded to study symbiont bacteria and/or archaea. We believe that the results of this study provide new insights into the complex nature of the rhizosphere and facilitate further studies on plants of importance for environmental remediation; this will be especially true for plants without extensive genome information.

## Methods

### Plant materials

Normal *Eichhornia crassipes* (NR) and purple root *Eichhornia crassipes* (PR) were provided by the Yunnan Institute for Ecological Agriculture^33^,^35^,^37^. The PR was bred through Gene Phenotype Induce Technique as described before^34^. The PR offspring were generated by tillering and bred normally. NR and PR plants and the offspring of PR plants were subjected to morphological measurements. The chloroplast *trnL-trnF* and *psbA-trnH* intergenic regions were amplified and sequenced as described previously to examine the genotype^57^.

### Heavy metal absorption

NR and PR of similar sizes were selected: approximately 60 ~ 80  long and average weight of 115 g. After culture in clear water for 14 days, plants were grown to 800 g and then transferred into water containing 2 mg/L nitrogen, 0.4 mg/L phosphate nutrient solution and CdCl_2_, and incubated at an ambient temperature of 24.8 °C with natural light. Three concentrations of Cd^2+^ ions were used: low (0.01 mg/L, Class V quality of surface water, defined as heavily polluted water according to the China National Standard GB 3838-2002), medium (0.1 mg/L) and high (1 mg/L). Water samples were taken at 1, 2, 4, 6, 8, 10, 12, 14, 16, and 30 days from three biological replicates. The heavy metal content at each point was determined by atomic absorption spectrometry method.

### RNA extraction and RNA-seq

Total RNAs were extracted from 2 g root tissue samples using the RNA Prep Pure Plant Kit according to the manufacturer’s protocol (Tiangen). RNA extracts from five randomly-selected individual plants of the same kind were pooled equally. Sequencing libraries were constructed following the NEBNext® mRNA Library Prep Master Mix Set for Illumina® protocol (NEB). Briefly, the polyA+ mRNA in the total mRNA samples were isolated using RNA Purification Beads (NEB), and the RNA-seq library was sequenced using Illumina HiSeq 2500 for 2 × 100 cycles. The raw sequencing datasets are available in the NCBI SRA database (accession number: SRP051408).

### *De novo* transcriptome assembly

Raw reads were processed to get clean reads by removing the adapter sequences and low quality reads. The *de novo* transcriptome assembly was performed using Velvet^58^ (version 1.2.10) and Trinity^59^,^60^ (release 20130225). After several trials, we selected a k-mer length of 51 for Velvet to produce optimal assembly results. The other parameters of Velvet remained at the default setting. Trinity was run with default parameters.

### Mapping

The non-redundant assembled contigs from NR and PR were merged and used as reference sequences. Bowtie^61^ (version 0.12.7) and FANSe2^30^ mapping software were used to align short reads. The 100 nt sequencing reads were split into two 50 nt reads before mapping. The options used for Bowtie mapping were set to -v 2 -M --best --strata. Reads were mapped to transcripts with the FANSe2 algorithm using the options –E2 –I0 –M0 –B1 –U0.

### Analysis of differentially expressed genes (DEGs)

The R statistical package software edgeR (Empirical analysis of Digital Gene Expression in R) was used to quantify differential gene expression^62^. Local false discovery rates (FDR) were calculated using the Benjamini-Hochberg (BH) method. FDR < 0.01 and |log_2_fold-change (log_2_FC)|≥3 were used to identify significant differences in transcript expression. The BLAST2GO program was used to obtain GO annotations^24^. WEGO software was used to determine the distribution of gene functions at the macroscopic level^63^. Functional enrichment analysis including Gene Ontology and KEGG pathways were performed for the identified DEGs.

### PCR-DGGE

DNA from 2g root samples was purified using the RaPure Plant DNA Kit (Magen Biotech, China) according to the manufacturer’s protocol. DNA extracted from five randomly selected individual plants of the same kind were equally pooled. ITS sequences were amplified with the primers NSA3 (AAACTCTGTCGTGCTGGGGATA) and NLC2 (GAGCTGCATTCCCAAACAACTC)^64^. A 50-μl reaction volume containing 25 μl DreamTaq Green PCR Master Mix (2×) (Thermo Scientific) was used. PCR conditions were as follows: 5 min at 94 °C for initial denaturation; followed by 30 cycles of 1 min at 94 °C for denaturation, 30 s at 55 °C for annealing, and 1 min at 72 °C for extension. The DGGE analysis was performed using an 8% polyacrylamide gel with a 30% to 60% urea gradient. The target bands were excised from the gel and the PCR product was purified and sequenced. The ITS sequence was analyzed using the nucleotide blast program at NCBI database.

### Quantitative real-time PCR

Real-time PCR was used to quantify the amount of *Fusarium* ITS2 sequence in each sample. The DNA (10 ng) of each sample was mixed with 0.001 ng reference gene (human GAPDH cDNA). The *Fusarium* ITS2 sequence was amplified with the primer pair qFs (5′-CAATCCCTGTTGGTTTCTTTTCC) and qFa (5′-CACCTCGTTACTGGTAATCGTCG). Quantitative real-time PCR experiments were performed in a Bio-Rad Mini Opticon Real-time PCR System. Expression ratios were calculated from cycle threshold values using the 2^−△△CT^ method.

### High-throughput sequencing of fungal rDNA ITS and data analysis

Sequencing libraries were constructed using PCR primers designed to amplify the ITS region. Template libraries were generated by PCR with following primers: ITS1 (TCCGTAGGTGAACCTGCGC) and ITS4 (TCCTCCGCTTATTGATATGC) and ITS4 (TCCTCCGCTTATTGATATGC) and ITS5 (GTGAATCATCGAATCTTTGAAC)^65^. The sequencing adapters with barcodes were added to the libraries by PCR. The libraries were normalized and pooled, and automated cluster generation and sequencing was carried out using an Illumina MiSeq system. Quality filtering, and operational taxonomic unit (OTU) picking were performed using the Quantitative Insights Into Microbial Ecology (QIIME) toolkit v.1.5.0^66^. The sequences were then clustered using UCLUST^67^ to representative OTUs at a sequence similarity of 97%. OTUs were assigned a taxonomic unit using RDP classifier implementation of QIIME. The raw sequencing datasets are available in the NCBI SRA database (accession number: SRP051408).

### Analysis of *Fusarium* DEGs

*Fusarium verticillioides* has the closest and most fully sequenced genome to the rDNA ITS sequence obtained here (WGS Project: AAIM02, GenBank Assembly ID: GCA_000149555.1). Transcriptome data were mapped to the *F. verticillioides* genome using FANSe2 with parameters –E8–I0 –M0 –B1 –U0. DEGs were identified using edgeR and functionally annotated with Blast2GO.

## Additional Information

**Accession codes:** The raw sequencing datasets are available in the NCBI SRA database (accession number: SRP051408).

**How to cite this article**: Luo, B. *et al*. Revealing crosstalk of plant and fungi in the symbiotic roots of sewage-cleaning *Eichhornia crassipes* using direct *de novo* metatranscriptomic analysis. *Sci. Rep*. **5**, 15407; doi: 10.1038/srep15407 (2015).

## Supplementary Material

Supplementary Information

Supplementary Table S2

Supplementary Table S5

## Figures and Tables

**Table 1:** *De novo* transcriptome assembly results.

	NR	PR	NR	PR
Assembly algorithm	Trinity		Velvet	
Number of transcripts >300 bp	167600	91687	61151	63664
Total base pairs (Mb)	175	108	28	30
N50 (bp)	1543	1678	456	461
Average sequence length (bp)	1050	1180	473	473
Median sequence length (bp)	710	872	394	398
Max length (bp)	11760	12165	3805	3272
%GC	48%	46%	49%	48%
Contigs aligned to NCBI-nr* database	76%	70%	76%	77%

NR = normal *Eichhornia crassipes*, PR = purple root *Eichhornia crassipes*

*NCBI-nr database = NCBI non-redundant database.

**Table 2:** Enriched KEGG pathway annotations in DEG.

	KEGG pathway	Number of DEGs
Upregulated: PR vs. NR	Plant hormone signal transduction (ko04075)	133
	Plant-pathogen interaction (ko04626)	126
	Starch and sucrose metabolism (ko00500)	105
	Amino sugar and nucleotide sugar metabolism (ko00520)	52
	Pentose and glucuronate interconversions (ko00040)	50
	Cell cycle (ko04110)	43
	Phenylpropanoid biosynthesis (ko00940)	42
	Flavonoid biosynthesis (ko00941)	39
	Phenylalanine metabolism (ko00360)	33
	Pyrimidine metabolism (ko00240)	30
Downregulated: PR vs. NR	Plant-pathogen interaction (ko04626)	70
	Ribosome (ko03010)	63
	Plant hormone signal transduction (ko04075)	61
	Starch and sucrose metabolism (ko00500)	43
	Aminobenzoate degradation (ko00627)	30
	Polycyclic aromatic hydrocarbon degradation (ko00624)	29
	Phenylalanine metabolism (ko00360)	28
	Focal adhesion (ko04510)	28
	Limonene and pinene degradation (ko00903)	28
	Regulation of actin cytoskeleton (ko04810)	27
	Stilbenoid, diarylheptanoid and gingerol biosynthesis (ko00945)	27
	Methane metabolism (ko00680)	27
	Bisphenol degradation (ko00363)	27

NR = normal *Eichhornia crassipes* root, PR = drug-treated plants with purple roots.

**Table 3:** Upregulated DEGs in *Fusarium*.

Contig	logFC	logCPM	*P*-Value	FDR	GO
contig210	7.143416	21.0943	4.31E-11	4.91E-09	
contig198	7.127261	20.80303	4.61E-11	4.91E-09	
contig18	5.31604	15.16745	1.17E-07	8.30E-06	
contig153	5.137349	15.73439	2.29E-07	1.22E-05	GO:0006412: translation
contig26	4.299617	13.8524	7.74E-06	0.00033	GO:0003779: actin binding
contig62	3.400592	13.85279	0.00023	0.006991	GO:0046872: metal ion binding
contig91	3.086647	12.64809	0.000792	0.018746	GO:0033554: cellular response to stress

logFC = log_2_-fold-change; logCPM = average log_2_-counts-per-million.
