# Supplementary material for: Revealing crosstalk of plant and fungi in the symbiotic roots of sewage-cleaning Eichhornia crassipes using direct de novo metatranscriptomic analysis
Source: Sci Rep. 2015 Oct 16;5:15407. doi: 10.1038/srep15407 (PMC4607945; doi:10.1038/srep15407)
Supplement: Supplementary Information [file srep15407-s1.doc]

**Revealing crosstalk of plant and fungi in the symbiotic roots of sewage-cleaning *Eichhornia crassipes* using direct *de novo* metatranscriptomic analysis**

Bin Luo, Wei Gu, Jiayong Zhong, Ying Wang* and Gong Zhang*

**Supplementary Materials**

**Table S1**: Statistics of the meta-transcriptomic sequencing datasets.

|  | NR | PR |
| --- | --- | --- |
| Nunber of clean Reads (millon) | 48.1 | 28.6 |
| Total of bases(Gb) | 9.6 | 5.7 |
| Q20 | 98% | 98% |
| Q30 | 94% | 94% |
| %GC | 54% | 54% |

**Table S2**: Gene expression of all transcript contigs in NR and PR. The numbers are given in read counts.

[Separate Excel sheet]

**Table S3**: GO terms of DEGs carried out by fungi-like genes and plant-like genes. These data are illustrated in Figure 4B.

| GO term | fungi | plants | fungi fraction | plants fraction | fungi/plant |
| --- | --- | --- | --- | --- | --- |
| nucleoside binding | 140 | 341 | 0.163361 | 0.105638 | 1.546416 |
| catabolic process | 129 | 401 | 0.150525 | 0.124226 | 1.211708 |
| transmembrane transporter activity | 52 | 174 | 0.060677 | 0.053903 | 1.125659 |
| ion binding | 94 | 329 | 0.109685 | 0.101921 | 1.076179 |
| oxidoreductase activity | 111 | 403 | 0.129522 | 0.124845 | 1.037458 |
| hydrolase activity | 133 | 510 | 0.155193 | 0.157993 | 0.982277 |
| anatomical structure morphogenesis | 41 | 177 | 0.047841 | 0.054833 | 0.872496 |
| reproductive developmental | 25 | 128 | 0.029172 | 0.039653 | 0.735669 |
| Cell stimulus | 22 | 121 | 0.025671 | 0.037485 | 0.684841 |
| multicellular organismal development | 70 | 386 | 0.08168 | 0.119579 | 0.683067 |
| cellular developmental process | 15 | 92 | 0.017503 | 0.028501 | 0.614124 |
| secondary metabolic process | 25 | 166 | 0.029172 | 0.051425 | 0.567263 |

**Table S4**: Statistics of fungi taxonomy

| Samples | NR | DR | CR |
| --- | --- | --- | --- |
| number of Clean data reads | 260848 | 120258 | 180598 |
| number of Clean data bases | 65132071 | 30019310 | 45115176 |
| Kingdom | 1 | 1 | 1 |
| Phylum | 7 | 5 | 5 |
| Class | 28 | 21 | 21 |
| Order | 59 | 43 | 44 |
| Family | 99 | 74 | 72 |
| Genus | 152 | 118 | 122 |

**Table S5**: Normalized ITU of NR, PR and CR.

[Separate Excel sheet]


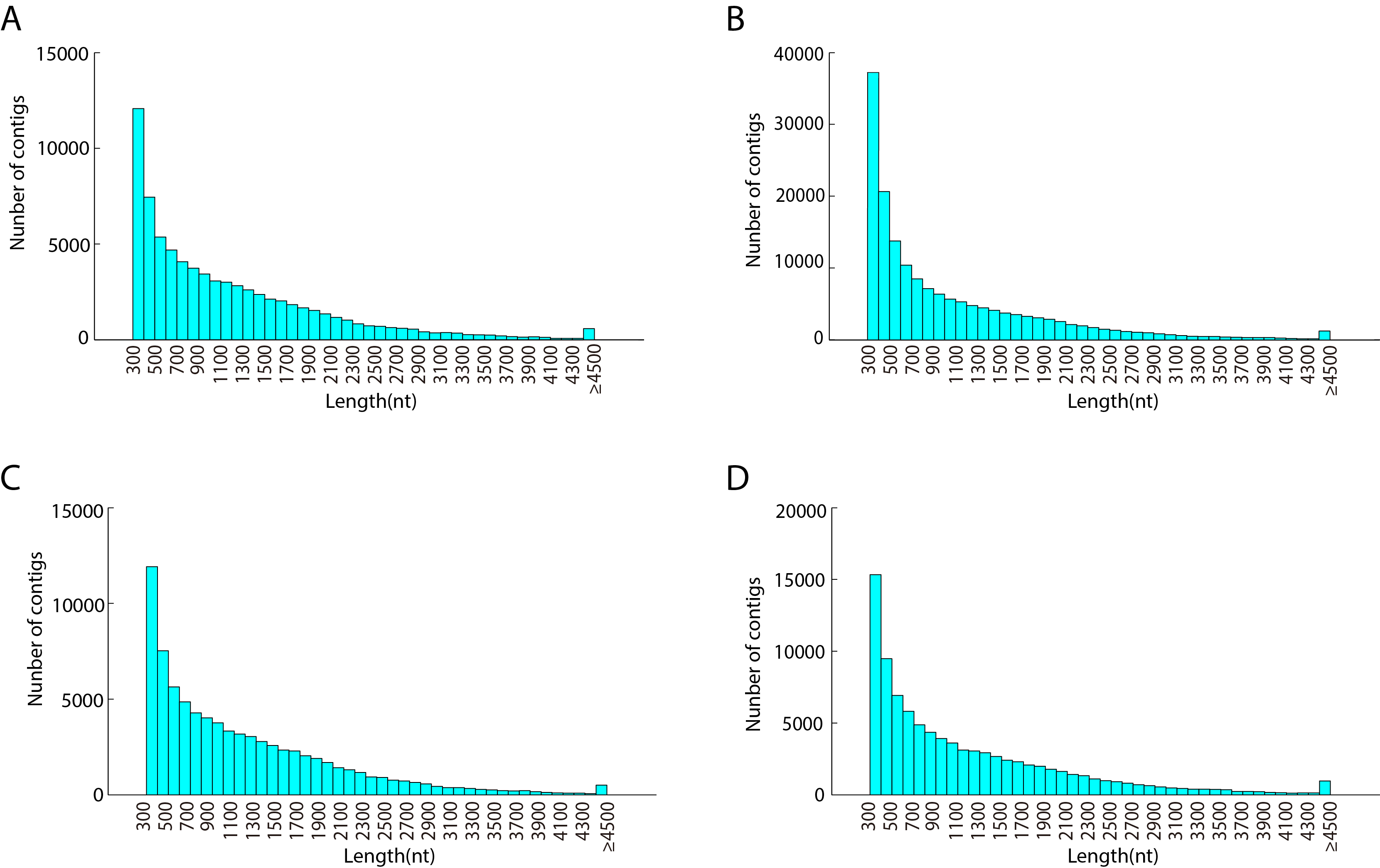


**Figure S1**: Length distribution of the *de novo* assembled contigs: (A) NR Velvet, (B) NR Trinity, (C) PR Velvet, (D) PR Trinity.

**Figure S2**: GO assignments under the GO term “Cell Cycle”. Terms with more than 20 assignments were marked as red.


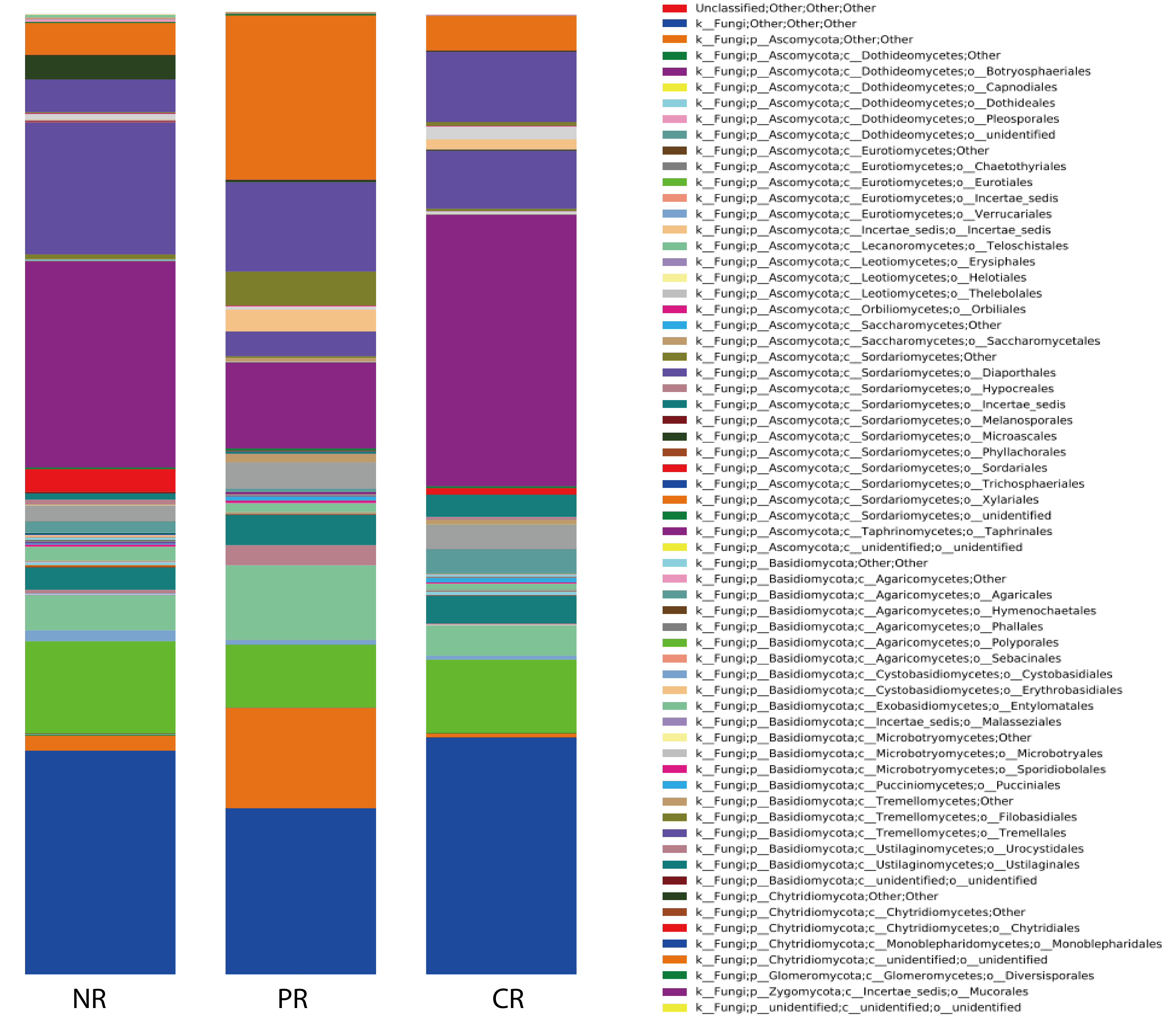


**Figure S3**: Fungi taxa assignments at class level, revealed by 18S ITS sequencing.
